# P-2139. Evaluation of a Multiplex Real-time PCR for the Detection and Differentiation of *Sporothrix schenckii* and *Sporothrix brasiliensis*

**DOI:** 10.1093/ofid/ofae631.2294

**Published:** 2025-01-29

**Authors:** Luisa F López Cano, Lalitha Gade, Anastasia P Litvintseva, Joseph Sexton

**Affiliations:** Centers for Disease control and prevention, Atlanta, Georgia; CDC, Lilburn, GA; Centers for Disease Control and Prevention, Atlanta, Georgia; Centers for Disease Control and Prevention, Atlanta, Georgia

## Abstract

**Background:**

*Sporothrix* spp is a thermally dimorphic fungi known to cause subacute or chronic subcutaneous lesions in humans and animals and is the cause of increasing public health concern due to spread of feline-associated cases.

**Figure 1.**
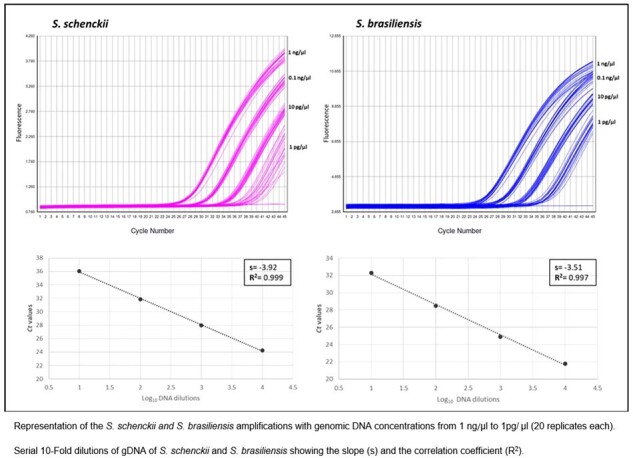
Limit of detection and genomic DNA standard curve of S. schenckii and S. brasiliensis

**Methods:**

A recently described Real-time PCR assay targeting the β-tubulin gene to detect and differentiate two related species, *S. schenckii* and *S. brasiliensis*, was evaluated and adapted by adding lambda primers and probe for internal control amplification. Isolates, fresh tissues and suspected sporotrichosis Formalin-Fixed, Paraffin Embedded (FFPE) tissues from humans and animals were received in the Mycotic Diseases Branch laboratory at the U.S. Centers for Disease Control and Prevention (CDC) for the routine fungal identification by conventional PCR and sequencing, using the primers D1/D2, ITS5/ITS4 and ITS3/ITS4. Ten-fold serial dilutions from 1 ng/μl to 1 fg/μl of DNA were tested with 20 replicates to establish limit of detection (LOD), and DNA from four fresh tissues extracted on three different days was used to assess reproducibility.

**Table 1.**
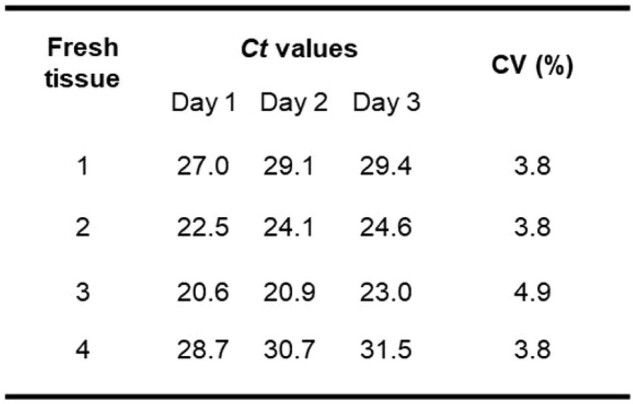
Ct values and coefficient of variation (CV) of the reproducibility test using fresh tissues on three different days

**Results:**

The assay was tested with 55 *S. brasiliensis,* 19 *S. schenckii*, and 85 isolates from other clinically relevant fungi, showing 100% concordance with sequencing methods. The test was also able to amplify *S. schenckii* DNA from 10 fresh tissues, which were previously confirmed by culture and sequencing as *S. schenckii* (100% sensitivity), with *Ct* values ranging from 19 to 38. Among 9 FFPE samples*, S. schenckii* was amplified in 6 samples (67% sensitivity) with *Ct* values ranging from 29 to 38. LOD was 1pg/μl of DNA. Results of the 20 replicates showed that at this concentration all the DNAs had a positive signal for both targets (fig 1). For reproducibility, the results showed consistent *Ct* values over the 3 days of the test with the four fresh tissue samples with a coefficient of variation (CV) of 4% (table 1).

**Conclusion:**

This multiplex Real-time PCR assay successfully detected DNA from the *S. brasiliensis* and *S. schenckii* isolates as well as *S. schenckii* from fresh and FFPE tissues. Our results demonstrate this molecular assay performs well and could be a helpful molecular tool to support rapid identification and differentiation at species level from cultures and primary human and animal specimens.

**Disclosures:**

All Authors: No reported disclosures

